# Impaired Cerebral Autoregulation in Parkinson's Disease: An Orthostatic Hypotension Analysis

**DOI:** 10.3389/fneur.2022.811698

**Published:** 2022-03-18

**Authors:** Yingqi Xing, Qing Li, Erhe Xu, Jingrong Zeng, Qiuping Li, Shanshan Mei, Yang Hua

**Affiliations:** ^1^Department of Vascular Ultrasonography, Xuanwu Hospital, Capital Medical University, Beijing, China; ^2^Beijing Diagnostic Center of Vascular Ultrasound, Beijing, China; ^3^Center of Vascular Ultrasonography, Beijing Institute of Brain Disorders, Collaborative Innovation Center for Brain Disorders, Capital Medical University, Beijing, China; ^4^Department of Neurology, Xuanwu Hospital, Capital Medical University, Beijing, China

**Keywords:** Parkinson's disease, cerebral autogregulation, orthostatic hypotension, transcranial color doppler, ultrasound

## Abstract

Orthostatic hypotension (OH) is an early non-motor manifestation of Parkinson's disease (PD). However, the underlying mechanism of hemodynamic changes in patients with PD and OH remains unclear. This study aimed to investigate the dynamic cerebral autoregulation changes in patients with PD with OH. Ninety patients with PD and 20 age- and sex-matched healthy controls (HCs) were recruited. The patients' non-invasive blood pressure (BP) and cerebral blood flow velocity were simultaneously recorded at supine and orthostatic positions during the active standing test (AST). Transfer function analysis was used to determine autoregulatory parameters including gain [i.e., damping effect of dynamic cerebral autoregulation (dCA) on the magnitude of BP oscillation] and phase difference (i.e., the time delay of the cerebral blood flow response to BP). Sixteen patients (17.8%) in the PD population were diagnosed with OH (PD-OH). The AST results were normal for 74 patients (82.2%) (PD-NOR). In the supine position, the PD-OH group had a lower phase degree than the PD-NOR group (50.3 ± 23.4 vs. 72.6 ± 32.2 vs. 68.9 ± 12.1, *p* = 0.020); however, no significant difference was found upon comparing with the HC group. In the orthostatic position, the normalized gain was significantly higher for the symptomatic OH group than for the asymptomatic OH group and HC group (1.50 ± 0.58 vs. 0.97 ± 0.29 vs. 1.10 ± 0.31, *p* = 0.019). A symptomatic OH in the PD population indicates an impaired cerebral autoregulation ability in the orthostatic position. Cerebral autoregulation tends to be impaired in the supine position in the OH population.

## Introduction

Parkinson's disease (PD) is a neurodegenerative disorder characterized by α-synuclein aggregation in the central and peripheral nervous systems, which could result in various non-motor manifestations (1), including autonomic and cognitive impairment. With active medical treatment of motor symptoms in patients with PD, non-motor symptoms gradually dominate and can be as disabling as motor symptoms.

Orthostatic hypotension (OH) is an early non-motor manifestation of PD. It is defined as a decrease of 20 mmHg in systolic blood pressure (SBP) or 10 mmHg in diastolic blood pressure (DBP) 3 min after standing (2). The OH indicates cardiovascular autonomic dysfunction and is characterized by dizziness, fatigue, sweating, and falling. It affects up to half the patients with PD and may result in functional disability and poor quality of life (3). Approximately 25% of patients with PD will develop cognitive impairment during their lifetimes (4). The OH and cognitive impairment are strongly correlated. A previous study identified that OH affects 41% of patients with dementia (5). Biogeau et al. evaluated older adults with OH and highlighted that cerebrovascular reactivity may be the key link between OH and cerebrovascular disorders (6). Further, Kario et al. reported silent cerebral infarction and white matter lesions detected by MRI in association with OH (7), which may negatively impact lifespan. A systematic review concluded that OH is associated with lower Mini-Mental State Examination (MMSE) scores (8). However, the mechanism underlying the association between OH and cognitive impairment remains unclear.

Dynamic cerebral autoregulation (dCA) refers to the capacity of adapting cerebral vasoconstriction and vasodilation to blood pressure (BP) fluctuations within a certain range to regulate and stabilize cerebral blood flow (9). A previous study established that impaired dCA is associated with cognitive decline and dementia in older patients (10). The sympathetic tone is impaired in patients with PD with OH. However, the degree of dCA impairment has not been established. We assume that impaired dCA results in cerebral hypoperfusion, which, in turn, accelerates cognitive impairment in patients with OH. However, there have been conflicting results and inconsistent conclusions by studies on dCA in PD. This study aimed to investigate the relationship between dCA and PD-associated OH.

## Materials and Methods

### Patients

The patients with PD were recruited from the neurology ward of the Capital Medical University Xuanwu Hospital in China from January 2021 to September 2021. The PD was diagnosed according to the United Kingdom (UK) Brain Bank criteria (11). All diagnoses were established by two independent neurologists. We included clinical diagnosis or probable diagnosis of PD. Age- and sex-matched healthy controls (HCs) without neurological disorders were also recruited from the general population. The exclusion criteria were as follows: atrial fibrillation, myocardial infarction, diabetes mellitus, poor temporal window prohibitive of transcranial doppler sonography (TCD) monitoring, communication difficulty, and severe systemic diseases, such as heart failure and pulmonary disorders, fever, and infectious diseases. Patients with focal lesions on MRI and CT examinations and intracranial or extracranial artery stenosis of >70% were also excluded. The baseline characteristics were recorded for all patients. We evaluated the Hoehn-Yahr (H-Y) stages and Unified Parkinson's Disease Rating Scale (UPDRS) scores and conducted ultrasound measurements of residual urine volume. The Non-Motor Symptoms Questionnaire (NMS-Quest) was used to screen for the presence of non-motor symptoms. We used the MMSE and Montreal Cognitive Assessment (MOCA) to assess cognitive impairment and the Hamilton Depression Scale (HAMD) to assess the severity of depression.

### Active Standing Test

All examinations were performed in a silent room free of distraction, with a maintained room temperature of 20–24°C. The participants were asked to avoid alcohol, caffeine, and nicotine and discontinue dopamine and vasoactive medications 24 h before the examination. After 10 min of relaxation in the supine position, they were asked to perform the AST, which involved lying in the supine position on the bed for 10 min and standing for 10 min (12). The symptoms were assessed during the entire procedure. The OH was diagnosed under one of the following conditions: (1) classic OH, decrease in SBP of ≥20 mmHg or DBP of ≥10 mmHg within 3 min of standing; or (2) delayed OH, decrease in SBP of ≥20 mmHg or DBP of ≥10 mmHg more than 3 min after standing (13). We distinguished neurogenic OH from non-neurogenic, OH using the neurogenic OH ratio based on the AST (14). Neurogenic OH was characterized by the Δheart rate (HR)/ΔSBP ratio of <0.492 during the AST. Based on these results, the participants were allocated to the orthostatic hypotension (PD-OH) and normal AST (PD-NOR) groups. According to the consensus statement on the definition of supine hypertension (15), supine hypertension was defined as SBP of ≥140 mmHg and/or DBP of ≥90 mmHg measured in the supine position for the PD-OH population.

### Symptom Assessment

To assess the presence of OH symptoms, we administered the Orthostatic Hypotension Questionnaire (OHQ). The OHQ consists of two parts: Orthostatic Hypotension Symptom Assessment (OHSA) and Orthostatic Hypotension Daily Activities Scale (OHDAS). The OHSA includes six domains of subjective feelings: (1) dizziness, lightheadedness, faintness, or impending “blackout”; (2) visual disturbance (blurring, scotoma, and tunnel vision); (3) weakness; (4) fatigue; (5) trouble concentrating; and (6) head/neck discomfort. The OHDAS evaluates the impact of OH symptoms on daily activities that require standing and/or walking for a brief or extended period of time within the past seven days. Each item is scored from 0 to 10, with 0 representing no symptoms and 10 representing the worst possible symptoms. The OHQ score is calculated as the average of the OHSA and OHDAS scores (16). We categorized the OH cases as symptomatic and asymptomatic based on the OHQ score and the AST BP measurement. Symptomatic OH was characterized by an OH diagnosis based on the AST and an OHQ score of more than zero, while asymptomatic OH was characterized by an OH diagnosis based on the AST and an OHQ score of zero.

### dCA Measurement

Baseline BP was measured at the brachial artery (Omron HBP-1300; Omron Healthcare, Kyoto, Japan) in the supine position. During a 10-min supine period, three BP readings were recorded. We used EMS-9D PRO (Delica Medical, Shenzhen, China) to simultaneously record non-invasive continuous BP (NIBP) and cerebral blood flow velocity (CBFV) in both the supine and standing positions during the entire procedure. The recorded NIBP (input signals) and CBFV (output signals) were used to calculate the cerebral autoregulation parameter based on the transfer function analysis (TFA) (17), which was based on Fourier decomposition of input and output signals into sines and cosines in the frequency domain. With the assumption of linear correlation, it quantifies how much NIBP was reflected in the CBFV. In addition to this, the regulator between NIBP and CBFV was indicated as cerebral autoregulation. The computer output parameters included phase shift, absolute gain (cm/s/mmHg), normalized gain (%/mmHg), coherence at very low frequency (0.02–0.07 Hz), low frequency (0.07–0.2 Hz), and high frequency (0.2–0.5 Hz). Phase shift could be the representation of the time delay of the CBFV response to NIBP, and a phase shift of 0 meant that there was no time delay of the CBFV response to NIBP. The gain represented the damping effect of dCA on the magnitude of BP oscillation. The absolute gain represented the absolute changes in NIBP and CBFV, whereas normalized gain represented a relative change in CBFV and NIBP regardless of baseline values of NIBP and CBFV. Cerebral autoregulation parameters were calculated with the assumption of linearity. However, the statistical reliability might be affected due to the presence of unrelated noise in reality. Coherence would approach one if the TFA systems were highly linear (18). For the frequency domain, we evaluated within a very low-frequency range (0.02–0.07 Hz), which was considered to reflect the most relevant real-time dynamic dCA behavior (19). Usually, we only estimated dCA parameters if the coherence is within 0.02–0.07 Hz was >0.6.

Meanwhile, a low phase and a high gain represented impaired dCA. An NIBP was measured using a servo-controlled plethysmograph at the middle finger. Two 2-MHz transcranial Doppler probes were placed over the temporal window and fixed with an adjustable head frame. Continuous CBFV was measured in the left and right middle cerebral arteries (MCAs) at a depth of 50–60 mm using the EMS-9D PRO (Delica Medical, Shenzhen, China). The exhaled CO_2_ was monitored using a nasal cannula connected to the EMS-9D. All procedures were performed by a professional ultrasound doctor.

### Experimental Design and Statistical Analysis

This research was designed as a single-center cross-sectional case-control study. Normally distributed continuous variables were expressed as mean ± standard deviation, continuous variables with skewed distribution were expressed as medians (interquartile ranges), and categorical variables were expressed as numbers (percentages). For equivalent variables with a normal distribution, the independent Student's *t*-test was used to compare the two groups. Bonferroni corrections were used to compare multiple groups. Receiver operating characteristic (ROC) analysis was performed to identify the cutoff value. The areas under the curve (AUCs), optimal threshold values, sensitivity, and specificity were calculated. Statistical significance was defined as a two-sided *p*-value of <0.05, and the confidence intervals (CIs) were set at 95%. The statistical analyses were performed using IBM SPSS (version 22.0) and GraphPad Prism (version 6.01).

### Standard Protocol Approvals, Registrations, and Patient Consent

This study was approved by the board of the ethics committee of the Capital Medical University Xuanwu Hospital. All participants voluntarily took part in the research with informed consent.

## Results

### Demographics

In total, 90 patients with PD (mean age, 58.8 ± 10.8 years; 55 men and 35 women) were recruited in this study (Supplementary Figure 1, Study Flow Chart), and 20 healthy volunteers (mean age, 61.6 ± 5.9 years; 10 men and 10 women) were recruited as controls. There were no differences in body mass index (BMI), BP, or HR between the groups. The demographic information of the participants is provided in Table 1. The OH was diagnosed in 16 patients (17.8%), including eight patients with symptomatic OH and eight with asymptomatic OH, according to the OHQ assessment.

**Table 1:** Demographic feature between each group.

	**PD (*N* = 90)**	**HC (*N* = 20)**	***P***
Sex (male)	55 (61.1)	10 (50)	0.361
Age	58.8 ± 10.8	61.6 ± 5.9	0.265
BMI	24.4 ± 4.4	23.2 ± 4.6	0.276
SBP (mmHg)	121 ± 16	115 ± 18	0.141
DBP (mmHg)	68 ± 10	64 ± 9	0.103
MAP (mmHg)	86 ± 11	85 ± 11	0.714
HR (bpm)	71 ± 10	74 ± 10	0.228

*BMI, body mass index; SBP, systolic blood pressure; DBP, diastolic blood pressure; MAP, mean blood pressure; HR, heart rate*.

### dCA Parameters in PD and HCs

Transcranial doppler monitoring of the MCA and beat-to-beat non-invasive BP recordings were performed for all participants in the supine and orthostatic positions. The dCA parameters and BP readings throughout the procedures are listed in Table 2.

**Table 2:** Cerebral autoregulation parameter during supine and orthostatic position.

		**PD (*N* = 90)**	**HC (*N* = 20)**	***P***
Supine
	BP power (mmHg^2^)	120.0 ± 95.2	116.3 ± 62.9	0.869
	CBFV power (cm^2^/s^2^)	117.0 ± 81.4	133.1 ± 88.8	0.433
	Gain (cm/s·mmHg)	0.71 ± 0.33	0.82 ± 0.29	0.172
	Gain (%/mmHg)	1.22 ± 0.46	1.28 ± 0.48	0.602
	Phase (deg)	68.6 ± 31.9	68.9 ± 12.1	0.967
	Coherence	0.63 ± 0.13	0.67 ± 0.05	0.179
	SBP (mmHg)	121 ± 16	115 ± 18	0.141
	DBP (mmHg)	68 ± 10	64 ± 9	0.103
	MAP (mmHg)	86 ± 11	85 ± 11	0.714
	HR (bpm)	71 ± 10	74 ± 10	0.228
	PSV (cm/s)	88 ± 25	92 ± 17	0.498
	EDV (cm/s)	39 ± 14	40 ± 9	0.761
	MV (cm/s)	55 ± 17	58 ± 11	0.453
	Et-CO_2_	39.2 ± 2.6	38.2 ± 3.4	0.145
Orthostatic
	BP power (mmHg^2^)	145.3 ± 119.7	179.7 ± 71.9	0.220
	CBFV power (cm^2^/s^2^)	184.2 ± 107.9	142.7 ± 78.4	0.107
	Gain (cm/s·mmHg)	0.59 ± 0.26	0.68 ± 0.21	0.151
	Gain (%/mmHg)	1.18 ± 0.45	1.10 ± 0.31	0.452
	Phase	59.5 ± 24.9	51.2 ± 11.9	0.150
	Coherence	0.68 ± 0.13	0.72 ± 0.04	0.178
	SBP 3 min (mmHg)	117 ± 20	117 ± 15	0.999
	DBP 3min (mmHg)	73 ± 15	68 ± 11	0.162
	MAP 3 min (mmHg)	88 ± 15	84 ± 8	0.251
	HR 3 min (bpm)	83 ± 13	80 ± 11	0.340
	PSV 3 min (cm/s)	80 ± 23	88 ± 15	0.141
	EDV 3 min (cm/s)	35 ± 13	38 ± 6	0.317
	MV 3 min (cm/s)	50 ± 16	55 ± 8	0.177
	Et-CO_2_	38.5 ± 2.5	38.0 ± 4.2	0.483

*BP, blood pressure; CBFV, cerebral blood flow velocity; SBP, systolic blood pressure; DBP, diastolic blood pressure; MAP, mean blood pressure; HR, heart rate; PSV, peak systolic velocity; EDV, end diastolic velocity; MV, mean velocity; Et-CO_2_, end-tidal carbon dioxide*.

As shown in Table 2, the phase and gain were equally distributed among the patients with PD and HCs in the supine and orthostatic positions. In addition, the BPs were not different in the supine and orthostatic positions.

### Demographic Features in Patients With PD With or Without OH

In total, 16 patients (17.8%) had OH, of whom eight were symptomatic. General characteristics, such as sex, BMI, hypertension, disease duration, and motor symptoms, were equally distributed between the PD-NOR and PD-OH groups. The use of medications, such as antihypertensives and levodopa (or daily equivalent dose), did not differ in the groups. The MMSE and MOCA scores were also not significantly different. The patients in the PD-OH group were older than those in the PD-NOR group (63.7 ± 7.7 vs. 57.7 ± 11.1 years, *p* = 0.041). Further details have been provided in Table 3. In the supine position, the SBP and mean arterial pressure (MAP) were higher in the PD-OH group than in the PD-NOR group (131 ± 23 vs. 118 ± 14 mmHg, *p* = 0.050; 92 ± 14 vs. 85 ± 11 mmHg, *p* = 0.029), whereas HR was lower in the PD-OH group than in the PD-NOR group (67 ± 6 vs. 72 ± 11 bpm, *p* = 0.015). Supplementary Figure 2 shows the MAP and mean velocity (MV) of the MCA at each moment in patients with or without OH. The MAP was decreased in the PD-OH group (*p* = 0.006). However, MV remained stable throughout the procedure (*p* = 0.10). Further details are provided in Supplementary Table 1.

**Table 3:** Demographic feature of orthostatic hypotension (OH) in patients with Parkinson's disease (PD).

	**PD-NOR (*N* = 74)**	**PD-OH (*N* = 16)**	***P***
Sex (male)	44 (59.5)	11 (68.8)	0.489
Age	57.7 ± 11.1	63.7 ± 7.7	0.041
BMI	23.9 ± 3.3	26.6 ± 7.3	0.165
Hypertension	24 (32.4)	4 (25.0)	0.560
Disease duration (year)	3.8 ± 3.9	4.3 ± 3.2	0.677
Tremor	30 (40.5)	8 (50.0)	0.487
Akinesia	57 (79.2)	11 (68.8)	0.368
REM sleep disorder	30 (40.5)	7 (43.8)	0.813
Use of medication			
AHD	20 (27.0)	4 (25.0)	0.739
ACEI/ARB	4 (5.4)	2 (12.5)	
Beta blocker	1 (1.4)	0	
CCB	12 (16.2)	2 (12.5)	
Diuretic	3 (4.1)	0	
LEDD (mg/d)	386 ± 298	448 ± 299	0.453
Residue urine volume (ml)	32.6 ± 66.3	51.3 ± 63.2	0.305
H-Y stage			0.308
1	15 (20.3)	4 (25.0)	
2	36 (48.6)	6 (37.5)	
3	20 (27.0)	4 (25.0)	
4	1 (1.4)	1 (6.3)	
5	2 (2.7)	0	
UPDRS scale	47.7 ± 27.6	49.9 ± 29.6	0.785
Non-motor scale	8.1 ± 6.6	9.5 ± 9.4	0.488
Motor scale	9.6 ± 7.4	10.4 ± 7.1	0.699
NMSS scale	33.5 ± 33.9	46.5 ± 50.3	0.208
HAMD scale	8.1 ± 7.6	6.5 ± 7.9	0.450
MOCA	21.9 ± 5.8	22.0 ± 5.3	0.949
MMSE	26.9 ± 3.2	26.7 ± 2.6	0.816
Symptomatic OH	0	8 (50.0)	<0.001
Supine hypertension	0	2 (12.5)	0.032

*REM, rapid eye movement; AHD, antihypertensive drugs; ACEI, angiotensin-converting enzyme inhibitor; ARB, angiotensin receptor blockers; CCB, calcium channel blocker; LEDD, levodopa equivalent daily dose; H-Y, hoehn-yahr; UPDRS, unified Parkinson's disease rating scale; NMS-Quest scale, non-motor symptoms questionnaire; HAMD, hamilton depression scale; MOCA, montreal cognitive assessment; MMSE, mini-mental state examination*.

### dCA Parameters in Patients With PD With or Without OH and HCs

As shown in Table 4, the PD-OH group had a lower phase degree than the PD-NOR group in the supine position (50.3 ± 23.4 vs. 72.6 ± 32.2 vs. 68.9 ± 12.1, *p* = 0.020). However, no significant difference was found between the phase degrees of the PD-OH and HC groups. The gain was equally distributed across the groups in the supine and orthostatic positions.

**Table 4:** Cerebral autoregulation parameter in patients with PD with or without OH.

		**PD-NOR (*N* = 74)**	**PD-OH (*N* = 16)**	**HC (*N* = 20)**	***P***
Supine					
	BP power (mmHg^2^)	120.1 ± 99.1	119.6 ± 78.1	116.3 ± 62.9	0.986
	CBFV power (cm^2^/s^2^)	97.1 ± 81.8	99.6 ± 129.5	145.9 ± 103.8	0.119
	Gain (cm/s·mmHg)	0.72 ± 0.25	0.76 ± 0.36	0.82 ± 0.29	0.346
	Gain (%/mmHg)	1.30 ± 0.42	1.21 ± 0.46	1.28 ± 0.48	0.756
	Phase (deg)	72.6 ± 32.2^*^	50.3 ± 23.4^*^	68.9 ± 12.1	0.020
	Coherence	0.63 ± 0.14	0.62 ± 0.19	0.67 ± 0.06	0.459
	Et-CO_2_	38.7 ± 2.2	38.5 ± 0.7	38.3 ± 3.4	0.780
Orthostatic					
	BP power (mmHg^2^)	142.2 ± 117.1	159.5 ± 134.5	179.7 ± 71.9	0.405
	CBFV power (cm^2^/s^2^)	91.6 ± 102.2	96.1 ± 134.3	142.7 ± 78.4	0.149
	Gain (cm/s·mmHg)	0.63 ± 0.22	0.75 ± 0.57	0.68 ± 0.21	0.313
	Gain (%/mmHg)	1.17 ± 0.44	1.24 ± 0.52	1.10 ± 0.31	0.470
	Phase (deg)	61.3 ± 24.8	51.3 ± 24.7	51.2 ± 11.9	0.104
	Coherence	0.69 ± 0.16	0.67 ± 0.05	0.72 ± 0.06	0.528
	Et-CO_2_	38.4 ± 2.4	38.1 ± 3.5	38.0 ± 4.2	0.836

* *Compared PD-NOR group and PD-OH group, adjusted p < 0.05*.

*BP, blood pressure; CBFV, cerebral blood flow velocity; Et-CO_2_, end-tidal carbon dioxide*.

### dCA Parameters of Symptomatic Patients With OH

**Figure 1:**
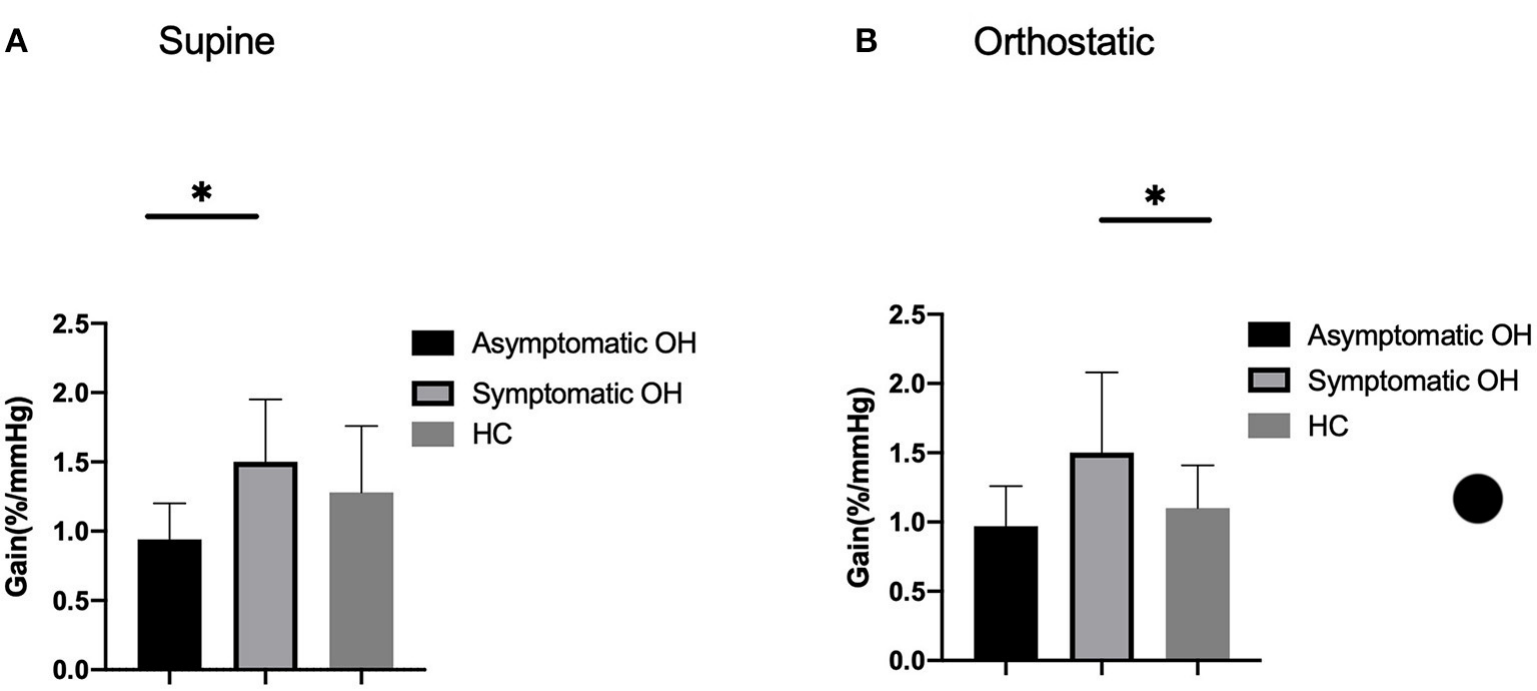
Outcome of cerebral autoregulation. Column bars represent normalized gain during **(A)** supine position and **(B)** orthostatic position. Lower gain indicates more effective cerebral autoregulation. *p < 0.05.

**Figure 2:**
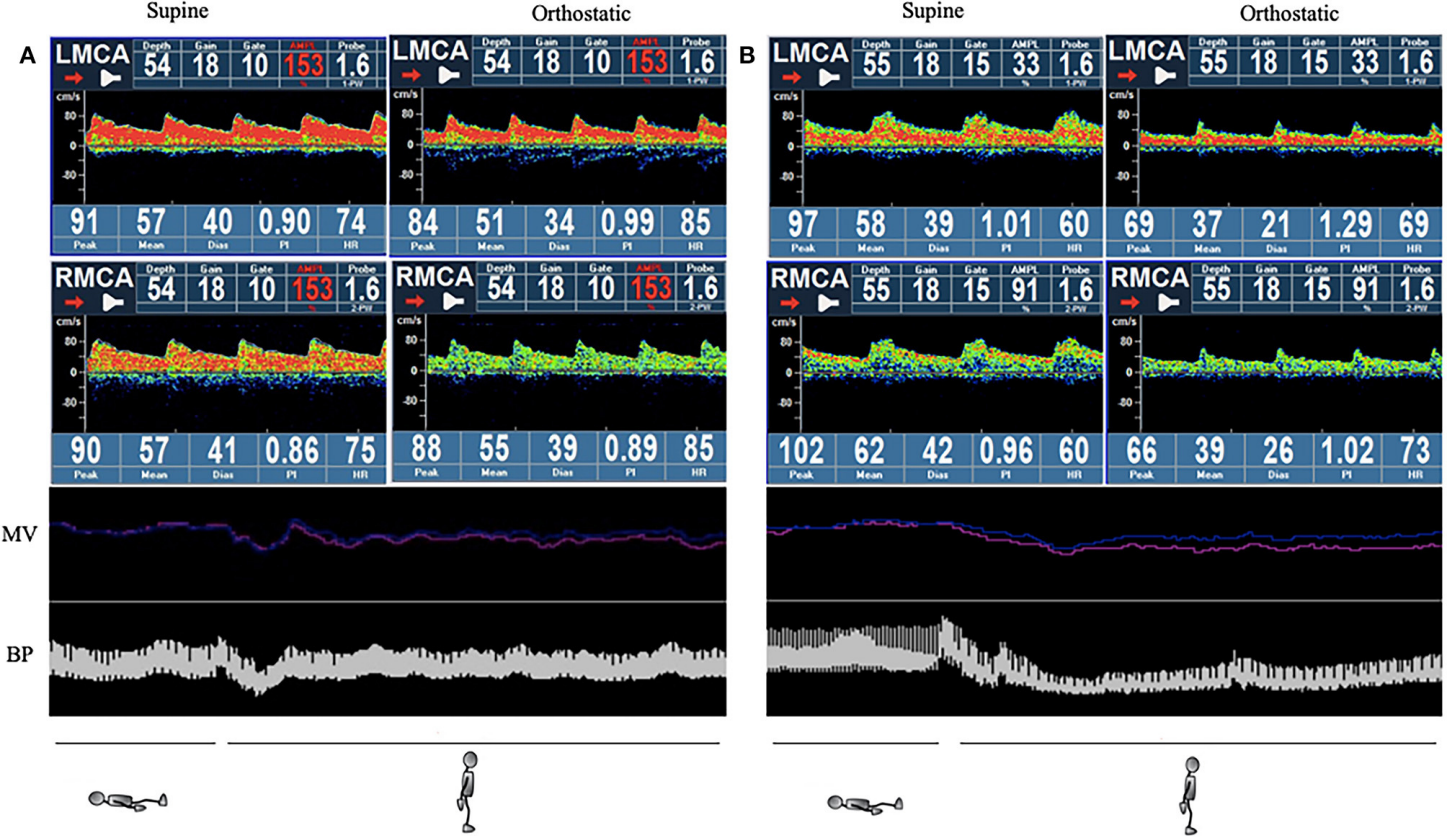
Example of simultaneous monitoring of non-invasive continuous blood pressure (NIBP) and mean velocity (MV) during the active standing test (AST). The red line indicates left side and the blue indicates right side in the MV section. **(A)** Healthy control: normal blood pressure (BP) drops and recovers quicky to normal levels without MV change. **(B)** Symptomatic orthostatic hypotension (OH) group: BP and MV drop considerably, and recovery is impaired. NIBP, non-invasive continuous blood pressure; MV, mean velocity; BP, blood pressure; OH, orthostatic hypotension.

The cerebral autoregulation parameters for each group are presented in Table 5 and Figure 1. In the supine position, the symptomatic OH group had a higher normalized gain than the asymptomatic patients with OH. However, the HC group did not show a significantly different normalized gain (1.50 ± 0.45 vs. 0.94 ± 0.26 vs. 1.28 ± 0.48, *p* = 0.046). In the orthostatic position, the normalized gain was significantly higher for the symptomatic OH group than the asymptomatic OH and HC groups (1.50 ± 0.58 vs.0.97 ±0.29 vs. 1.10 ±0.31, *p* = 0.019). The MAP and MV were not different for the groups in the supine position. However, the symptomatic OH group had a lower MAP than the asymptomatic OH group and the HCs in the orthostatic position (67 ± 20 vs. 82 ± 22 vs. 84 ± 8 mmHg, *p* = 0.033). The MV was significantly decreased in the symptomatic OH group (40 ± 4 vs. 50 ± 20 vs. 55 ± 8). Figure 2 shows that the MV and MAP changes during a postural change in the symptomatic OH group and the HC group.

**Table 5:** Cerebral autoregulation parameter in symptomatic patients with OH.

		**Asymptomatic OH (*N* = 8)**	**Symptomatic OH (*N* = 8)**	**HC (*N* = 20)**	***P***
Supine					
	MAP (mmHg)	92 ± 17	92 ± 11	85 ± 11	0.263
	MV (cm/s)	51 ± 27	56 ± 13	58 ± 11	0.589
	BP power (mmHg^2^)	142.8 ± 74.9	96.4 ± 78.9	116.3 ± 62.9	0.909
	CBFV power (cm^2^/s^2^)	78.3 ± 89.3	95.6 ± 79.3	145.9 ± 103.8	0.192
	Gain (cm/s·mmHg)	0.57 ± 0.26^*^	0.98 ± 0.35^*^	0.82 ± 0.29	0.030
	Gain (%/mmHg)	0.94 ± 0.26^*^	1.50 ± 0.45^*^	1.28 ± 0.48	0.046
	Phase (deg)	70.8 ± 27.6	57.3 ± 27.6	68.9 ± 12.1	0.328
	Coherence	0.68 ± 0.06	0.66 ± 0.04	0.67 ± 0.06	0.779
	Et-CO_2_	38.8 ± 2.2	38.2 ± 0.9	38.2 ± 3.4	0.868
Orthostatic					
	MAP 3 min (mmHg)	82 ± 22	67 ± 20^#^	84 ± 8^#^	0.033
	MV 3 min (cm/s)	50 ± 20	40 ± 4^#^	55 ± 8^#^	0.011
	BP power (mmHg^2^)	185.3 ± 114.7^*^	133.7 ± 155.1	179.7 ± 71.9	0.525
	CBFV power (cm^2^/s^2^)	107.6 ± 188.5	84.7 ± 29.5	142.7 ± 78.4	0.397
	Gain (cm/s·mmHg)	0.50 ± 0.15	0.73 ± 0.38	0.68 ± 0.21	0.145
	Gain (%/mmHg)	0.97 ± 0.29	1.50 ± 0.58^#^	1.10 ± 0.31^#^	0.019
	Phase (deg)	60.2 ± 10.8	51.1 ± 20.9	51.2 ± 11.9	0.293
	Coherence	0.68 ± 0.05	0.67 ± 0.04	0.72 ± 0.06	0.057
	Et-CO_2_	38.4 ± 2.6	37.9 ± 2.5	38.0 ± 4.2	0.955

* *Compared Asymptomatic OH and Symptomatic OH group, adjusted p < 0.05*.

# *Compared Symptomatic OH and HC group, adjusted p < 0.05*.

*MAP, mean blood pressure; MV, mean velocity; BP, blood pressure; CBFV, cerebral blood flow velocity; Et-CO_2_, end-tidal carbon dioxide*.

## Discussion

Our research showed dCA function preservation in all participants with PD. However, compared with the healthy population, dCA was associated with a risk of impairment in the PD-OH group in the supine position and was compromised in the symptomatic PD-OH subgroup in the orthostatic position.

Blood volume redistributes to the lower limbs and splanchnic organs when changing to a standing posture, resulting in reduced cardiac output which contributes to low cerebral perfusion (20). When autonomic nerve function is intact, a postural change produces a temporary decrease in BP with a quick return to normal levels. Cerebral autoregulation ensures that cerebral blood flow does not extensively fluctuate during this period. A previous study concluded that dCA was preserved in patients with PD (21). In our study, we compared the dCA of patients with PD and HCs and found no differences. In the supine position, the phase degree was lower in the PD-OH group than in the PD-NOR group, but the difference was not statistically significant. This suggests a tendency of impairment of dCA function in the PD-OH population. We analyzed the symptomatic OH subgroup and found compromised autoregulation in the orthostatic position. Most previous studies have only analyzed cerebral autoregulation in the supine position, which may fail to identify patients with impaired dCA in the upright position. We considered that impaired dCA would manifest during physiological stimulation as MAP sharply decreases and can hardly normalize in patients with OH. A prolonged BP recovery would induce decreased cerebral blood flow. Previous research suggested that a standing MAP of <75 mmHg is highly sensitive and specific for detecting symptomatic OH, indicating that a MAP of <75 mmHg is likely below the lower threshold for cerebral autoregulation in patients with PD (22). In our research, we subdivided the OH group according to the presence of symptoms and concluded that in patients with symptomatic OH, a cerebral autoregulation is impaired in the orthostatic position.

In patients with dysfunctional cerebral autoregulation, unstable flow through the distal capillary may injure the cerebral microcirculation. This, in turn, will damage the microvascular system and induce several downstream sequelae including the disruption of the blood-brain barrier, neuroinflammation, cerebral microbleeds, and white matter lesions. Previous studies have reported a strong correlation between small vessel disease with a decline in cognitive performance and executive function in older adults (23, 24). Growing evidence also suggests a relationship between impaired cerebral autoregulation and white matter hyperintensity (25, 26). White matter hyperintensity presents in up to 50% of patients with PD and has been associated with cognition and gait disorders (27). Impaired cerebral autoregulation may be a potential mechanism of cognitive dysfunction. In our research, we discovered that autoregulation is impaired in the orthostatic position in patients with PD with symptomatic OH. A previous study demonstrated that an upright posture exacerbated the deficits in executive function and memory, which are related to visuospatial function in the PD-OH population (28). Specific research about position-related cerebral autoregulatory changes in different cognitive domains has been designed by our research group.

Orthostatic hypotension (OH) presents in up to 41% of patients with dementia. Anang et al. followed 80 patients who are cognitively intact with PD for 4.4 years and found that the risk of developing dementia increased by 84% for each 10 mmHg drop in SBP during postural changes (29). Centi et al. (28) and Peralta et al. (30) reported a relationship between lower cognitive scores and OH in patients with PD. Their conclusions supported the hypothesis that changes in cerebral blood flow negatively affect cognition. Soennesyn et al. claimed that persistent OH was not predictive of cognitive or functional decline in individuals with mild-to-moderate dementia. So far, the relationship between OH and cognitive impairment remains controversial. In our research, we compared the MMSE and MOCA scores of the PD-OH and PD-NOR groups, but no differences were found. This may be due to the following reasons. First, we observed that the dysfunctional dCA in the symptomatic OH group was only presented in the upright position in our study. As a result of cerebral hypoperfusion, the patients may have had early stages of cognitive impairment that were not detected. Second, OH is related to a specific type of cognitive impairment. Previous research has suggested that PD with OH manifests poorer performance in sustained attention and visuospatial function (20). The MMSE and MOCA scores may be useful for selecting the cognitive impairment in the community phenomenon (31). Therefore, a comprehensive assessment tool would be needed for these specific patients. Third, this was a cross-sectional study, and the adverse effect of impaired cerebral autoregulation on cognitive function may have taken time to manifest. In future research, we plan to follow this patient group to assess their clinical outcomes and cognitive impairment.

Several limitations of this study should be clarified. First, this was a single-center case-control study. A larger cohort study should be conducted to confirm our findings. Second, the comprehensive assessment of cognitive impairment in patients with OH requires redesign. Although MMSE and MOCA are the most commonly used clinical cognitive function assessment methods, they could still not reflect the real status of some patients. Third, the active standing and head-up tilt (HUT) tests are used to detect OH, and previous studies have reported that they are better at predicting clinical outcomes even though the AST can simulate daily patient postures well (12). Our test choice may have led to an underestimation of OH incidence.

In conclusion, our research showed that in patients with PD with OH, there is an impaired tendency of autoregulation in the supine position. The symptomatic OH group demonstrated damaged cerebral autoregulation in the orthostatic position.

## Data Availability Statement

The raw data supporting the conclusions of this article will be made available by the authors, without undue reservation.

## Ethics Statement

The studies involving human participants were reviewed and approved by the board of the Ethics Committee of Capital Medical University Xuanwu Hospital. The patients/participants provided their written informed consent to participate in this study.

## Author Contributions

YX, QinL, EX, JZ, QiuL, and SM collected the data. QinL conducted data analysis and wrote the manuscript. YX and QinL put forward the study concepts and interpreted the results. YX and YH revised the manuscript and commented on the data. YX, QinL, and YH participated in the study design and manuscript revision. All authors have read and approved the final version of the manuscript.

## Conflict of Interest

The authors declare that the research was conducted in the absence of any commercial or financial relationships that could be construed as a potential conflict of interest.

## Publisher's Note

All claims expressed in this article are solely those of the authors and do not necessarily represent those of their affiliated organizations, or those of the publisher, the editors and the reviewers. Any product that may be evaluated in this article, or claim that may be made by its manufacturer, is not guaranteed or endorsed by the publisher.

## Supplementary Material

Supplementary Figure 1 Study flow chart.

Supplementary Figure 2 Mean arterial pressure (MAP) and mean velocity (MV) changes during postural changes between Parkinson's disease with orthostatic hypotension (PD-OH) and Parkinson's disease without orthostatic hypotension (PD-NOR) groups. **(A)** MAP changes at each moment during the active standing test (AST) between OH groups. **(B)** Changes in MV of MCA at each moment during the AST between OH groups. MAP, mean arterial pressure; MV, mean velocity; PD-OH, Parkinson's disease with orthostatic hypotension; PD-NOR, Parkinson's disease without orthostatic hypotension; MCA, middle cerebral artery.

Supplementary Table 1 Blood pressure and cerebral blood flow velocity in patients with or without OH.

Supplementary Table 2 MAP cutoff value for detecting symptomatic OH.
