# Supplementary figures and images for: Impaired Cerebral Autoregulation in Parkinson's Disease: An Orthostatic Hypotension Analysis
Source: Front Neurol. 2022 Mar 18;13:811698. doi: 10.3389/fneur.2022.811698 (PMC8971280; doi:10.3389/fneur.2022.811698)

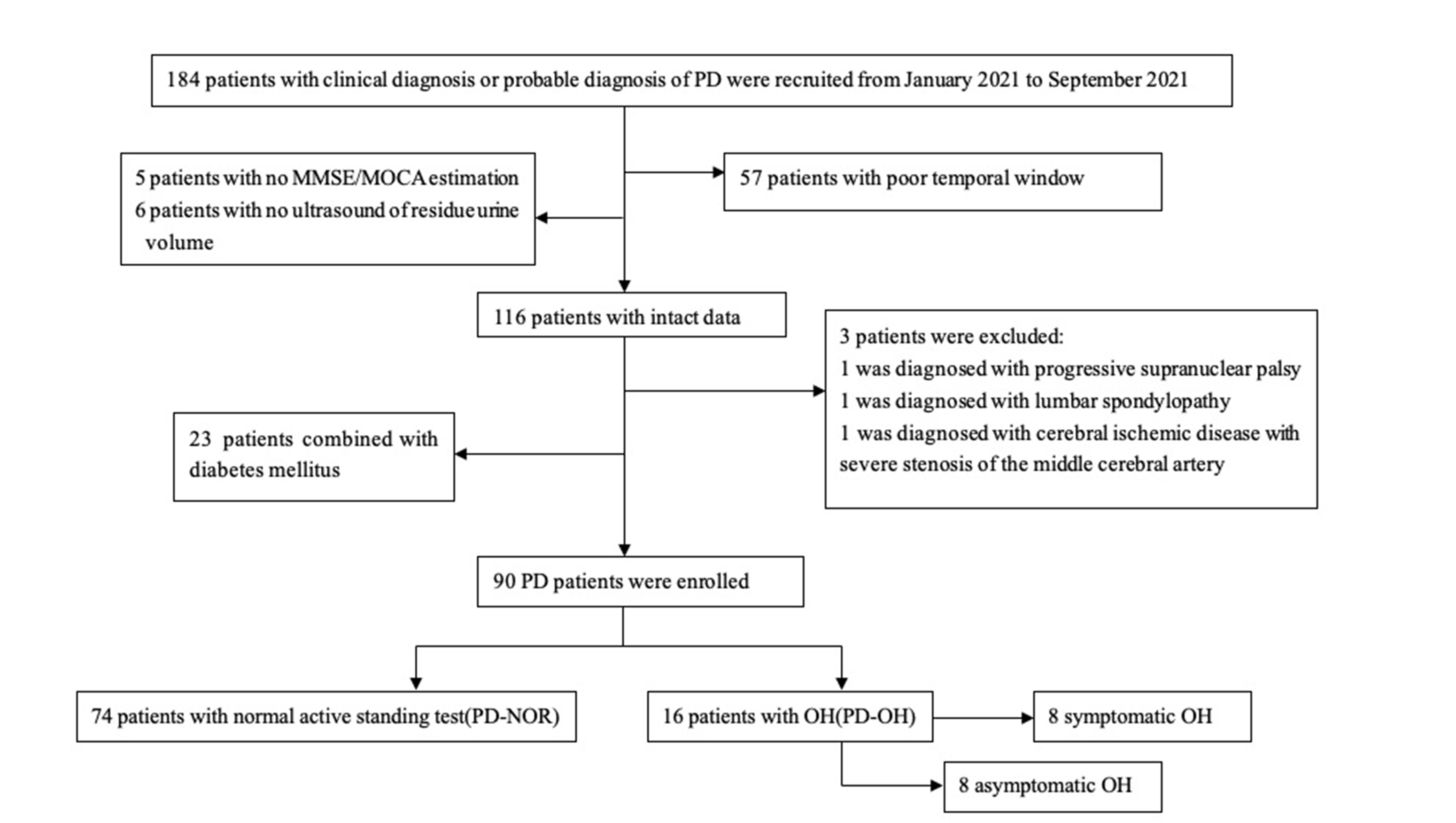

Supplement: Supplementary Figure 1 — Study flow chart. [file Image_1.JPG]

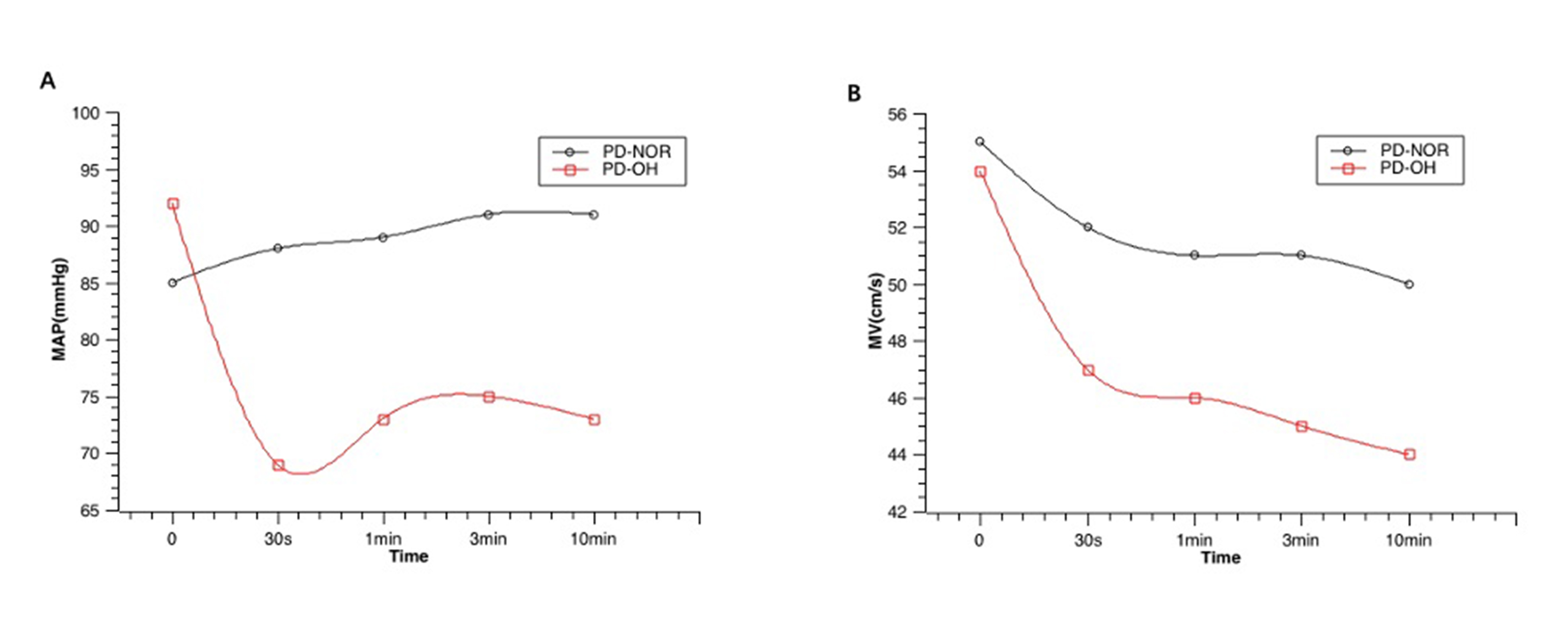

Supplement: Supplementary Figure 2 — Mean arterial pressure (MAP) and mean velocity (MV) changes during postural changes between Parkinson's disease with orthostatic hypotension (PD-OH) and Parkinson's disease without orthostatic hypotension (PD-NOR) groups. (A) MAP changes at each moment during the active standing test (AST) between OH groups. (B) Changes in MV of MCA at each moment during the AST between OH groups. MAP, mean arterial pressure; MV, mean velocity; PD-OH, Parkinson's disease with orthostatic hypotension; PD-NOR, Parkinson's disease without orthostatic hypotension; MCA, middle cerebral artery. [file Image_2.JPG]
