# Supplementary material for: Impaired Cerebral Autoregulation in Parkinson's Disease: An Orthostatic Hypotension Analysis
Source: Front Neurol. 2022 Mar 18;13:811698. doi: 10.3389/fneur.2022.811698 (PMC8971280; doi:10.3389/fneur.2022.811698)
Supplement: Supplementary Table 1 — Blood pressure and cerebral blood flow velocity in patients with or without OH. [file Table_1.docx]

| Supplementary Table1. Blood pressure and cerebral blood flow velocity in patients with or without OH | | | | |
| --- | --- | --- | --- | --- |
|  |  | PD-NOR(N=74) | PD-OH(N=16) | P |
| Supine |  |  |  |  |
|  | SBP(mmHg) | 118±14 | 131±23 | 0.050 |
|  | DBP(mmHg) | 68±10 | 71±12 | 0.363 |
|  | MAP(mmHg) | 85±11 | 92±14 | 0.029 |
|  | HR(bpm) | 72±11 | 67±6 | 0.015 |
|  | PSV(cm/s) | 88±22 | 88±34 | 0.957 |
|  | EDV(cm/s) | 39±13 | 37±14 | 0.484 |
|  | MV(cm/s) | 55±16 | 54±21 | 0.728 |
|  | Et-CO_2_ | 38.7±2.2 | 38.5±0.7 | 0.658 |
| Orthostatic |  |  |  |  |
|  | SBP30s(mmHg) | 118±18 | 98±31 | 0.028 |
|  | DBP30s(mmHg) | 73±12 | 55±17 | 0.001 |
|  | MAP30s(mmHg) | 88±13 | 69±21 | 0.003 |
|  | HR30s(bpm) | 84±13 | 79±14 | 0.245 |
|  | PSV30s(cm/s) | 83±21 | 79±30 | 0.580 |
|  | EDV30s(cm/s) | 36±14 | 31±14 | 0.164 |
|  | MV30s(cm/s) | 52±16 | 47±18 | 0.294 |
|  |  |  |  |  |
|  | SBP1min(mmHg) | 117±20 | 102±32 | 0.086 |
|  | DBP1min(mmHg) | 75±15 | 58±18 | <0.001 |
|  | MAP1min(mmHg) | 89±13 | 73±22 | 0.009 |
|  | HR1min(bpm) | 84±13 | 80±13 | 0.300 |
|  | PSV1min(cm/s) | 81±21 | 79±34 | 0.789 |
|  | EDV1min(cm/s) | 36±14 | 30±13 | 0.124 |
|  | MV1min(cm/s) | 51±16 | 46±19 | 0.330 |
|  |  |  |  |  |
|  | SBP3min(mmHg) | 120±16 | 104±30 | 0.037 |
|  | DBP3min(mmHg) | 76±12 | 59±19 | 0.004 |
|  | MAP3min(mmHg) | 91±12 | 75±22 | 0.010 |
|  | HR3min(bpm) | 84±13 | 81±13 | 0.066 |
|  | △HR_3_/△SBP_3_(beats/minute·mmHg) | 2.669±4.269 | 0.323±0.179 | <0.001 |
|  | PSV3min(cm/s) | 81±20 | 77±32 | 0.459 |
|  | EDV3min(cm/s) | 37±13 | 30±12 | 0.113 |
|  | MV3min(cm/s) | 51±15 | 45±18 | 0.247 |
|  |  |  |  |  |
|  | SBP10min(mmHg) | 122±15 | 103±26 | 0.029 |
|  | DBP10min(mmHg) | 76±12 | 57±16 | 0.002 |
|  | MAP10min(mmHg) | 91±12 | 73±19 | 0.006 |
|  | HR10min(bpm) | 84±13 | 82±16 | 0.544 |
|  | PSV10min(cm/s) | 80±21 | 77±37 | 0.641 |
|  | EDV10min(cm/s) | 35±13 | 28±13 | 0.069 |
|  | MV10min(cm/s) | 50±15 | 44±20 | 0.224 |
|  | Et-CO_2_ | 38.4±2.4 | 38.1±3.5 | 0.679 |

| Supplementary Table 2. MAP cutoff value for detecting symptomatic OH | | | | |
| --- | --- | --- | --- | --- |
|  | AUC | sensitivity | specificity | Youden index |
| MAP cutoff | 0.664 | 0.75 | 0.625 | 0.375 |
